# Unraveling 4‐Phenylbutyrate's Therapeutic Role in SLC6A1 Disorders: Pharmacochaperoning Over HDAC Inhibition

**DOI:** 10.1002/acn3.70430

**Published:** 2026-05-19

**Authors:** Melissa B. DeLeeuw, Karishma Randhave, Ekta Anand, Ziang (Debbie) Song, Wangzhen Shen, Jing‐Qiong Kang

**Affiliations:** ^1^ Department of Neurology Vanderbilt University Medical Center Nashville Tennessee USA; ^2^ Neuroscience Graduate Program TMNH Nashville Tennessee USA; ^3^ Vanderbilt Brain Institute TMNH Nashville Tennessee USA; ^4^ Vanderbilt University Nashville Tennessee USA; ^5^ Department of Pharmacology Vanderbilt University Medical Center Nashville Tennessee USA; ^6^ Vanderbilt Kennedy Center of Human Development Nashville Tennessee USA

**Keywords:** 4‐phenylbutyrate, autism, chaperone, endoplasmic reticulum, epilepsy, GABA transporter 1 (GAT‐1)

## Abstract

**Objective:**

Variants in *SLC6A1*, encoding the GABA transporter 1 (GAT‐1), cause epilepsy, autism spectrum disorder, and developmental delay via loss of GABA uptake, impaired trafficking, and ER retention. We previously found that 4‐Phenylbutyrate (PBA), an FDA‐approved drug, restores GABA uptake and reduces seizures in *SLC6A1*‐related disorders, prompting a phase I clinical trial (NCT04937062) primarily designed to evaluate safety and tolerability of Ravicti (PBA). However, its exact mechanism, pharmacochaperoning or histone deacetylase (HDAC) inhibition, remains unclear. This study compares PBA with pharmacochaperones and HDAC inhibitors to determine how it restores GAT‐1 function in the cell and mouse models.

**Methods:**

We evaluated the function for 32 *SLC6A1* variants and two representative mouse models at baseline and various treatment options using ^3^H GABA uptake assays. We evaluated the effect of PBA in comparison with other chaperone inducers or HDAC inhibitors on the mutant and the wild‐type GAT‐1 expression. Importantly, we evaluated the effect of chaperone inducers and HDAC inhibitors on GAT‐1 function and seizures in two representative knock‐in mouse models, *Slc6a1* ^*+/A288V*^ and *Slc6a1* ^*+/S295L*^, with EEG recordings.

**Results:**

PBA restored GABA uptake and GAT‐1 surface expression across all variants, and TUDCA mimicked the effects of PBA. HDAC inhibitors exhibited modest rescue in vitro but failed to restore GAT‐1 function or mitigate seizures in the knockin mice.

**Interpretation:**

PBA acts as a pharmacochaperone, not an HDAC inhibitor, to restore GAT‐1 function and reduce seizure burden in the diseased mice, supporting pharmacochaperoning as the major mechanism for rescuing *SLC6A1*‐related disorders.

**Abbreviations**
(ER): Endoplasmic Reticulum
(ERAD): ER‐associated degradation
(EYFP): enhanced yellow fluorescent protein
(GABA): γ‐aminobutyric acid
(GAT‐1): γ‐aminobutyric acid transporters
(HDAC): histone deacetylase
(HEK293T): human embryonic kidney
(PBA): 4‐phenylbutyrate
(SAHA): suberoylanilide hydroxamic acid
(SLC6): solute carrier
(SLC6A1): solute carrier family 6 member 1
(TUDCA): tauroursodeoxycholic acid

## 1 Introduction

Variants in *SLC6A1*, encoding GABA transporter 1 (GAT‐1), are implicated in developmental epileptic encephalopathies (DEEs), autism spectrum disorder, and intellectual disability [1, 2, 3]. GAT‐1 is essential for inhibitory neurotransmission, facilitating the reuptake of γ‐aminobutyric acid (GABA) from the synaptic cleft to maintain proper excitation/inhibition balance in the mammalian brain. Loss of function due to *SLC6A1* variants disrupts this balance and contributes to treatment‐resistant epilepsy and neurodevelopmental impairments, highlighting the urgent need for effective therapeutics [2, 4, 5]. In addition to mutations in genes that directly regulate GABAergic inhibition, such as *SLC6A1* and GABA_A_ receptor genes, prior studies have shown that impaired GABAergic inhibition also contributes to abnormal network excitability in several non‐GABA genetic epilepsies, including those associated with *CaV2.1* and *SCN1A* variants, reinforcing the need for targeted interventions that restore inhibitory tone [6, 7]. These findings also align with broader efforts to characterize seizure networks and seizure clustering driven by impaired GABAergic signaling [8].

A major pathogenic consequence of *SLC6A1* variants is loss of GABA uptake caused by reduced cell‐surface expression of GAT‐1 [2, 9, 10]. Protein misfolding and trafficking defects are widely recognized in neurodegenerative diseases but are increasingly implicated in early‐onset epilepsy and other childhood neurodevelopmental disorders [11]. Our prior work demonstrated that *SLC6A1* variants share mechanistic overlap with GABA_A_ receptor subunit variants, particularly in protein misfolding, ER retention, and trafficking impairments [10, 12, 13]. This suggests that therapeutic strategies targeting protein misfolding could be applied to *SLC6A1*‐mediated disorders.

4‐Phenylbutyrate (PBA) has been shown to rescue GAT‐1 function and mitigate seizures in both *Slc6a1* ^*+/S295L*^ and *Gabrg2* ^*+/Q390X*^ mice associated with epilepy [12, 14, 15]. Its preclinical efficacy in improving GAT‐1 function and reducing seizure burden motivated an ongoing phase I open‐label clinical trial evaluating the safety and tolerability of PBA (Ravicti) in individuals with *SLC6A1*‐related disorders (NCT04937062). While this study was not designed or powered to assess efficacy, clinical observations suggested drastic seizure improvement in some participants [16]. However, Ravicti remains costly and difficult to access until the clinical trial concludes, prompting patients to explore alternative treatments. Despite encouraging preclinical findings and preliminary clinical observations, the precise mechanism through which PBA restores GAT‐1 function has remained partially understood. PBA acts as both a pharmacochaperone and a histone deacetylase (HDAC) inhibitor, raising the critical question of whether its therapeutic effects are mediated by direct post‐translational protein rescue or epigenetic modulation of gene expression [14, 17, 18].

Butyrate, an HDAC inhibitor, has gained popularity as a low‐cost alternative to PBA, largely due to its structural similarity to PBA and accessibility. However, butyrate has not been mechanistically validated in *SLC6A1* disorders, raising concerns about whether HDAC inhibition alone is sufficient for GAT‐1 rescue. Our previous research has demonstrated that PBA directly rescues GABA uptake and restores GAT‐1 function in *SLC6A1* variants by stabilizing variant proteins and promoting their proper trafficking [12, 14, 19]. This dual mechanism distinguishes PBA from butyrate, suggesting its efficacy in *SLC6A1* disorders may rely more on pharmacochaperoning than epigenetic modulation. This distinction is critical for refining therapeutic strategies.

This study evaluates 32 *SLC6A1* loss‐of‐function missense variants using in vitro and ex vivo functional assays and knock‐in mouse models to determine the optimal mechanism and compound for restoring GAT‐1 function. By distinguishing whether pharmacochaperoning or HDAC inhibition drives PBA's efficacy, these findings provide critical mechanistic insight into *SLC6A1* variant‐mediated disorders, informing ongoing clinical trials and future therapeutic strategies.

## 2 Materials and Methods

### 2.1 *SLC6A1* Variants Knockin Mouse Models

Slc6a1^+/A288V^ and Slc6a1^+/S295L^ mouse models, housing, and husbandry were previously characterized in our studies [2, 3, 15]. Mice were housed 2–5 per temperature‐controlled cage on a 12‐h light–dark cycle with standard chow, water ad libitum, and nestlet enrichment.

### 2.2 cDNAs for Coding GABA Transporter 1

The plasmid cDNA encoding enhanced yellow fluorescent protein (YFP)‐tagged rat GAT‐1 was previously described [2, 12, 15, 19]. All GAT‐1 variants utilized were generated with site‐directed mutagenesis and confirmed through DNA sequencing.

### 2.3 *SLC6A1* Variant Selection and Phenotypic Annotation

The *SLC6A1* variants used in this study were not collected specifically for the present study. Variant information was obtained from previously published literature or communicated by caregivers for the purpose of molecular characterization of transporter function. In all cases, only the specific genetic variant and high‐level phenotypic descriptors were provided, without personal identifiers, access to medical records, or protected health information. These variants were incorporated into an existing cDNA library developed over multiple years for functional studies. No direct interaction with human subjects occurred, and no identifiable human data were collected; therefore, institutional review board approval was not required.

### 2.4 Polyethylenimine (PEI) Transfection

HEK293T cells were transfected using a polyethylenimine (PEI)‐based transfection protocol, as previously described [19].

### 2.5 Radioactive ^3^H‐Labeled GABA Uptake Assay

The radioactive ^3^H‐labeled GABA uptake assay has been described in our previous studies [2, 9, 19]. Due to the scale of experimental conditions tested, control values (pcDNA and CI‐966 treated) were reused across certain experiments, as appropriate. These shared controls were performed concurrently and included in each assay to allow direct comparison across multiple variants and treatment conditions. This intentional design ensured consistency while minimizing experimental variability.

### 2.6 Western Blot Analysis of GAT‐1 Protein in Different Degrees of Glycosylation

Western blot (WB) was conducted as previously described [9, 19]. As shown in prior work, GAT‐1 protein appears as three distinct bands, reflecting different glycosylation states: A high molecular weight mature form and two lower molecular weight immature forms retained in the ER [9].

### 2.7 Drug Administration in Vitro and in Vivo

For in vitro drug administration, stock solutions of each compound were prepared in DMSO and applied for 24 h at concentrations established in prior studies: PBA [2 mM], TUDCA [100 μM], butyrate [2 mM], SAHA [2.5 μM], and salubrinal [15 μM] [15, 17, 20, 21, 22]. For in vivo administration of PBA, dose and duration followed our established *SLC6A1* mouse protocols [15]. For butyrate administration in mice, the most efficacious dose was determined from previous studies [15, 23]. Mice of both sexes at 2–4 months of age were dosed with the drug (100 mg/kg, i.p.) or vehicle for 7 days. Butyrate solution was prepared by dissolving butyrate in 0.9% normal saline and then titrating equimolecular amounts of butyric acid (Sigma, Madrid, Spain) and 1 Mol potassium hydroxide to pH 7.4. The working solution was stored at 4°C.

### 2.8 Synchronized Video‐Monitoring EEG Recordings and Analysis

EEG electrode implantation, recovery, and recording procedures were conducted as previously described [24]. Our study examined both male and female animals, and similar findings were observed across sexes. However, sex was not a primary variable in the experimental design or analysis. EEG recordings were analyzed using Clampfit 11.4. Raw traces were imported, and a threshold search was performed to identify seizure‐like events, with thresholds individually determined from each mouse's baseline EEG. Events were detected between defined trigger and rejection markers; those outside this window or not meeting criteria were excluded. Burst analysis was conducted to assess seizure duration, intra‐burst interval, and frequency. Rhythmic bursts within the 4–8 Hz (θ wave) range were identified and quantified. All animal procedures were approved by the Vanderbilt University Medical Center Institutional Animal Care and Use Committee (IACUC) and were conducted in accordance with institutional guidelines for humane care and use of laboratory animals.

### 2.9 Statistical Analysis

Data were expressed as mean ± SEM. For comparisons evaluating baseline function, data were normalized to WT GAT‐1, which was arbitrarily set to 1 in each experiment. For drug treatment experiments, statistical comparisons were performed within each variant by comparing untreated (vehicle‐treated) and treated (drug‐treated) conditions for that variant using unpaired *t*‐tests; no shared control group was used across variants. One‐way analysis of variance (ANOVA) was used where appropriate, followed by post hoc Dunnett's multiple comparison tests. For direct pairwise comparisons (e.g., WT vs. variant or vehicle vs. drug), paired or unpaired *t*‐tests were used. All statistical analyses were performed in GraphPad Prism version 10.4.2 (La Jolla, CA), with significance set at *p* < 0.05.

## 3 Results

### 3.1 Partial to Complete Loss of Function Underlies a Wide Range of Neurodevelopmental Disorders Associated With *SLC6A1* Variants

**FIGURE 1:**
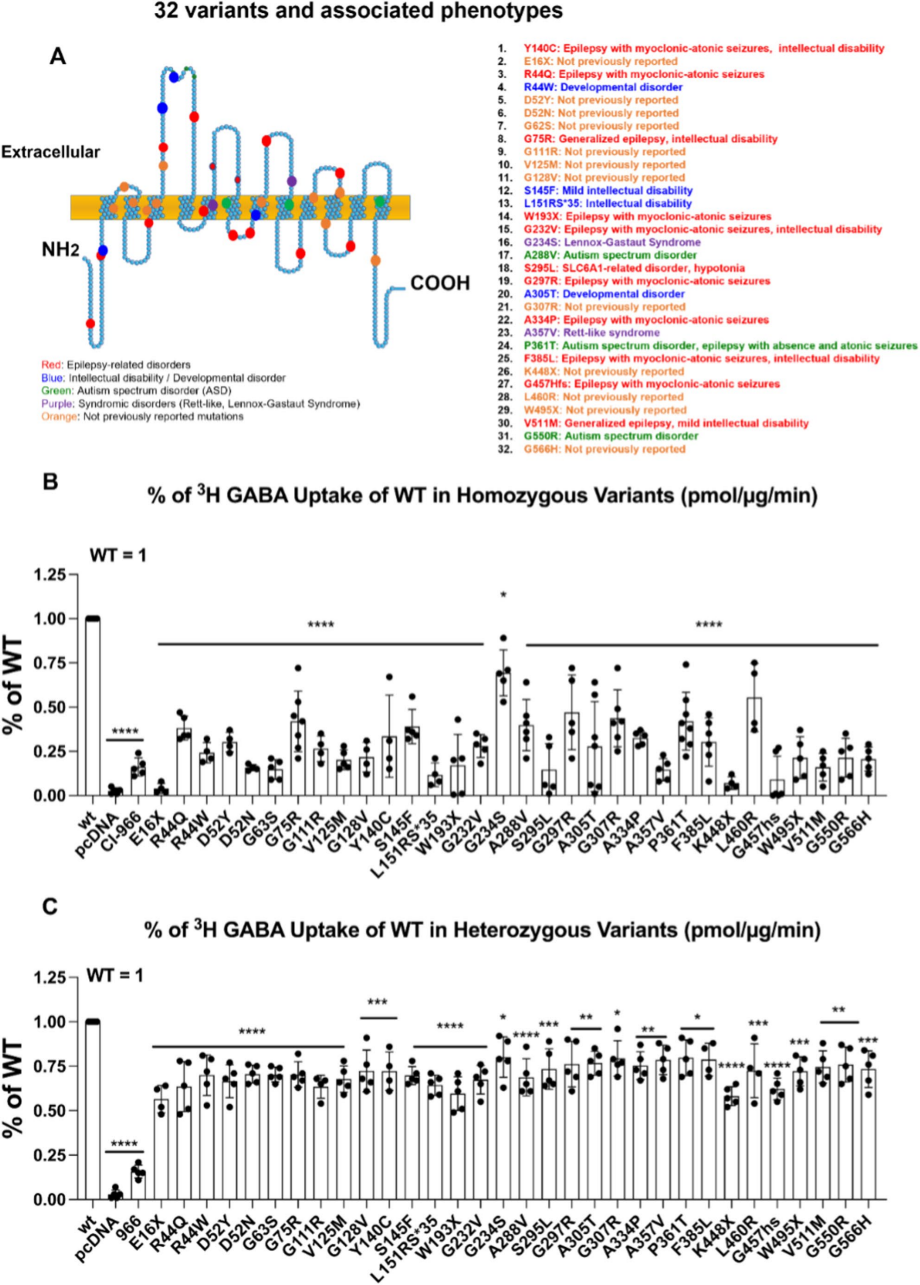
Disease Phenotypes of SLC6A1 Variants and Loss of Function of the mutant protein. (A) Topological schematic of GABA transporter 1 (GAT‐1) depicting 32 SLC6A1 variants mapped across the transmembrane domains. Variants are color‐coded based on their associated clinical phenotypes. The spectrum of missense and nonsense variants includes both previously characterized and novel variants, highlighting the genetic and phenotypic heterogeneity observed in SLC6A1‐related disorders. Phenotypic features reflect general clinical characteristics reported in the literature or by caregivers and are provided for contextual illustration only; overlapping phenotypes are common in SLC6A1‐related disorders. (B) Baseline GABA uptake function in homozygous SLC6A1 variants compared to wild‐type (WT). HEK293T cells were transfected with either WT or variant GAT‐1 constructs, and uptake was measured using a 3H GABA uptake assay on a liquid scintillation counter. Uptake values are normalized to WT, with pcDNA serving as a negative control and WT treated with CI‐966 [50 μM] as a functional reference. **All 32 variants exhibited a significant reduction in GABA uptake compared to WT; WT vs. G234S (*p = 0.0102), WT vs. all other variants (****p < 0.0001), as determined by one‐way ANOVA with Dunnett's post hoc test for multiple comparisons. An empty vector (pcDNA) was included as a negative control, and WT treated with CI‐966 [50 μM], a known GAT‐1 inhibitor, served as a functional reference. Values are expressed as mean ± SEM. (C) Baseline GABA uptake function in heterozygous SLC6A1 variants compared to WT. HEK293T cells were transfected with a 1:1 ratio of WT and variant GAT‐1 constructs, and uptake was measured using a 3H GABA uptake assay on a liquid scintillation counter. Uptake values are normalized to WT. Statistical analysis was performed using one‐way ANOVA, and Dunnett's multiple comparisons test was used to compare each variant to WT. *p < 0.05: G234S, P361T, F385L; **p < 0.01: G297R, A305T, G307R, A357V, G550R; ***p < 0.001: Y140C, S295L, A334P, L460R, V511M, G566H; ***p < 0.0001: All remaining variants.

Pathogenic variants in *SLC6A1* have been identified in patients with a broad range of neurodevelopmental and epilepsy disorders [2, 25, 26]. To systematically characterize the impact of *SLC6A1* missense variants, we selected 32 patient‐identified variants that span the full‐length topology of the GAT‐1 protein. Variants were color‐coded based on their associated clinical phenotypes, illustrating the genetic heterogeneity underlying *SLC6A1* variant‐mediated disorders (Figure 1A).

We first assessed GABA uptake in HEK293T cells transfected with either wild‐type (WT) or variant GAT‐1 constructs. In the “homozygous” condition, where only the variant protein was expressed, all 32 variants showed significantly reduced uptake relative to WT (Figure 1B). Several variants resulted in near‐complete loss of uptake, while others retained partial activity. CI‐966 [50 μM], a known GAT‐1 inhibitor, reduced uptake to the level of the negative control (pcDNA), validating the assay's sensitivity. We next evaluated GABA uptake in the “heterozygous” condition as seen in patients by co‐transfecting WT and variant GAT‐1 cDNA at a 1:1 ratio (0.25 μg each). All variants exhibited significantly reduced GABA uptake relative to WT (Figure 1C). The remaining GABA uptake activity averaged 56.5% to 80.2% of WT levels, corresponding to a 19.8% to 43.5% loss of function.

### 3.2 PBA and TUDCA Restored GABA Uptake in *SLC6A1* Variants

**FIGURE 2:**
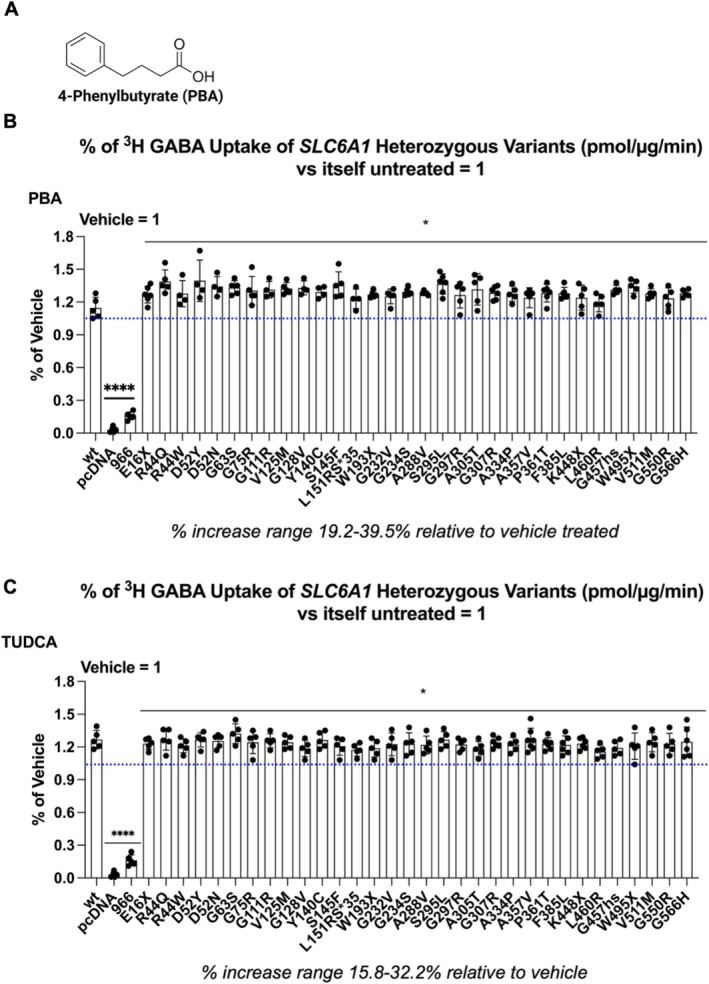
Pharmacochaperoning effect Restored GABA Uptake of the mutant protein in *SLC6A1* Variants in both cell and mouse models. (A) Chemical structure of 4‐phenylbutyrate (PBA). (B) ^3^H GABA uptake assay in heterozygous *SLC6A1* variants after PBA treatment ([2 mM], 24 h). HEK293T cells expressing GAT‐1 variants were treated with PBA, and uptake was quantified using a liquid scintillation counter. PBA significantly increased GABA uptake function across all 32 variants, restoring function to or above WT levels. Controls included empty‐vector (pcDNA) and WT treated with CI‐966 [50 μM], a known GAT‐1 inhibitor. Statistical analysis was performed using multiple unpaired t tests to compare PBA treatment to untreated across each condition, with a mean increase of 22.2% ± 4.9%. (C) ^3^H GABA uptake assay in heterozygous *SLC6A1* variants after TUDCA treatment ([100 μM], 24 h). HEK293T cells expressing GAT‐1 variants were treated with TUDCA, and uptake was quantified using a liquid scintillation counter. TUDCA significantly increased GABA uptake function across all 32 variants, although the magnitude of rescue was slightly lower than with PBA. Controls included empty‐vector (pcDNA) and WT treated with CI‐966 [50 μM], a known GAT‐1 inhibitor. Statistical analysis was performed using multiple unpaired *t*‐tests to compare TUDCA treatment to untreated across each condition, with a mean increase of 16.4% ± 4.6%.

Pathogenic SLC6A1 variants disrupt GAT‐1 function through a combination of impaired trafficking, altered protein folding, and reduced transporter activity, although the relative contribution of ER retention vs. intrinsic functional deficits may vary across variants [12, 15]. We previously showed that PBA, known as a pharmacochaperone as well as an HDAC inhibitor, restores the function of GAT‐1 variants (Figure 2A). To determine whether pharmacochaperoning or HDAC inhibition underlies PBA's therapeutic effects, we tested the ability of both PBA and TUDCA, as both have pharmacochaperoning effects to restore GABA uptake and GAT‐1 function in a heterozygous model. TUDCA, a known pharmacochaperone, was selected as a mechanistic comparison to PBA because it has been shown to reduce ER stress and stabilize misfolded proteins in various neurodegenerative diseases [20]. Given that GAT‐1 trafficking deficits arise from protein misfolding and ER retention, TUDCA provides an important benchmark for evaluating whether PBA functions through a similar post‐translational rescue mechanism.

HEK293T cells were transfected with a 1:1 ratio of WT and variant GAT‐1 cDNA to mimic patients' heterozygous conditions. Cells were treated for 24 h with either PBA [2 mM] or TUDCA [100 μM], followed by a ^3^H GABA uptake assay. Uptake was normalized to each variant's own vehicle‐treated baseline (self = 1) to observe the effect of each compound independently. PBA treatment resulted in a significant increase in uptake across all 32 variants, with the increase in uptake ranging from 19.2%–39.5% relative to the untreated baseline (Figure 2B). Statistical comparisons using multiple unpaired *t*‐tests confirmed that the uptake increase following PBA treatment was significant across nearly all variants (*p < 0.05*). TUDCA also significantly increased GABA uptake in every variant tested, with uptake ranging from 15.8%–32.2% relative to untreated (Figure 2C). Although the magnitude of rescue was slightly lower than that observed with PBA, the variant‐by‐variant response pattern was highly consistent, indicating a likely shared mechanism of action.

### 3.3 HDAC Inhibitors Had a Modest and Variable Upregulation of GABA Uptake in *SLC6A1* Variants

**FIGURE 3:**
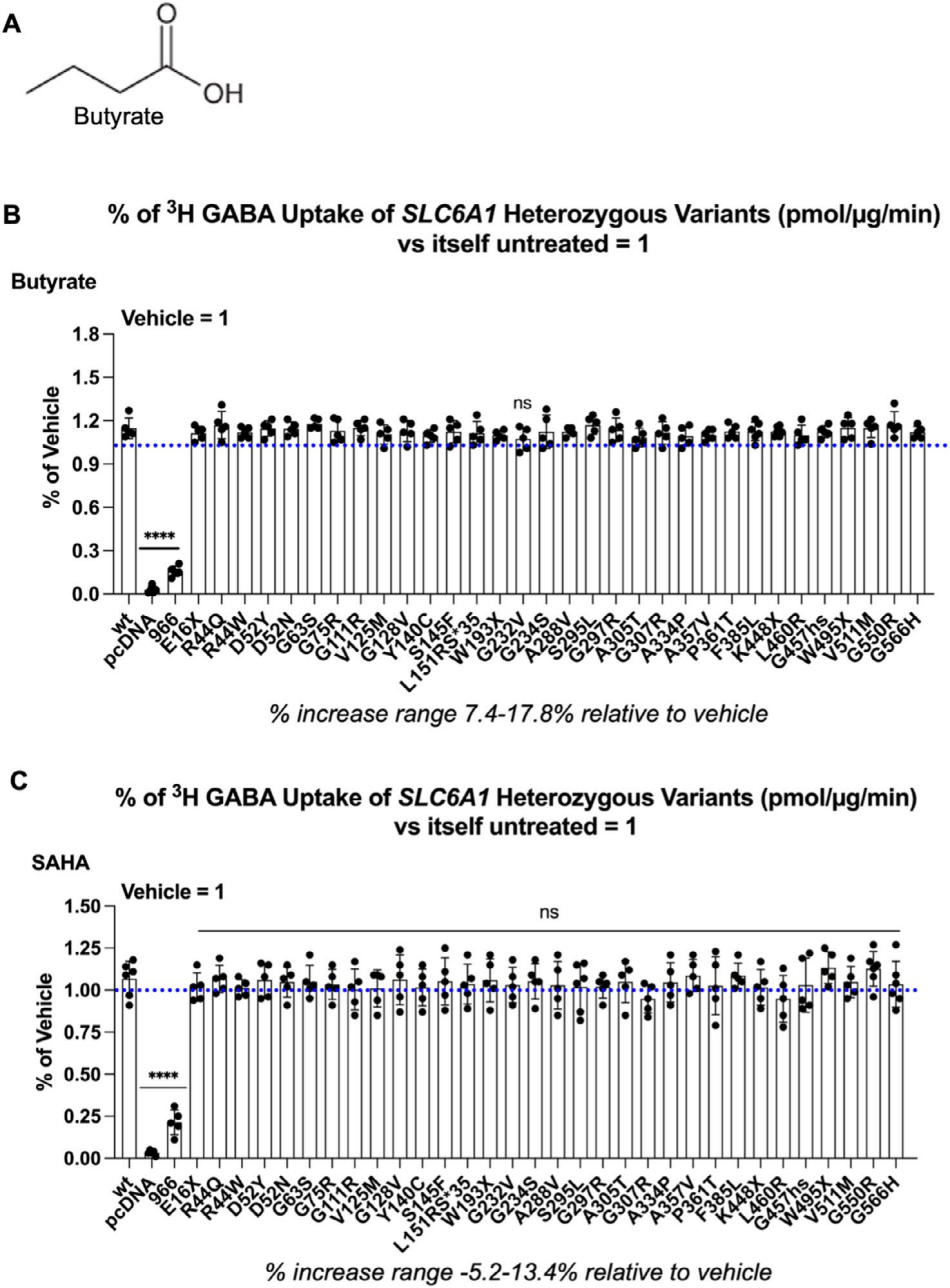
Limited Rescue of GABA Transport Function by HDAC Inhibitors in *SLC6A1* Variants. (A) Chemical structure of butyrate. (B) ^3^H GABA uptake assay in heterozygous *SLC6A1* variants after butyrate treatment ([2 mM], 24 h). HEK293T cells expressing GAT‐1 variants were treated with butyrate, and uptake was quantified using a liquid scintillation counter. Butyrate significantly increased GABA uptake function in the majority of variants, with a mean increase of 6.7% ± 4.1%. Statistical analysis was performed using multiple unpaired t tests to compare butyrate treatment to untreated across each condition. Only G232V did not reach statistical significance (ns), whereas all other variants showed statistically significant increases in GABA uptake following butyrate treatment. Variants reaching significance include (****p < 0.0001): G63S, K448X, pcDNA, CI‐966; (***p < 0.001): E16X, R44W, S295L; (**p < 0.01): R44Q, D52Y, D52N, G111R, G128V, Y140C, G297R, G307R, G550R, G566H, WT; (*p* < 0.05): G75R, G117R, V125M, S145F, L151RS35, W193X, G234S, A305T, A334P, A357V, P361T, F385L, L460R, V511M, W495X. Only G232V did not reach statistical significance. (C) ^3^H GABA uptake assay in heterozygous *SLC6A1* variants after SAHA treatment (2.5 μM, 24 h). HEK293T cells expressing GAT‐1 variants were treated with SAHA, and uptake was quantified using a liquid scintillation counter. SAHA significantly increased GABA uptake only in pcDNA (****p < 0.0001). No other variant reached statistical significance. Mean increase in uptake was **−**1.2% ± 3.7%. Controls included empty‐vector (pcDNA) and WT treated with CI‐966 [50 μM], a known GAT‐1 inhibitor. Statistical analysis was performed using multiple unpaired *t*‐tests comparing SAHA treatment to untreated across each condition. For each *SLC6A1* variant, statistical comparisons were performed between untreated and butyrate‐treated conditions within the same variant. Untreated values are not displayed in the graph for visual clarity but were used exclusively as matched controls for statistical analysis.

While PBA robustly restored GABA uptake across all *SLC6A1* variants tested, it remained unclear whether HDAC inhibition alone could elicit similar functional rescue. Butyrate, a short‐chain fatty acid with structural similarity to PBA, is a widely used HDAC inhibitor and has been proposed as a low‐cost alternative for patients seeking accessible treatment options (Figure 3A). To address this, we evaluated the effects of two chemically distinct HDAC inhibitors, butyrate and SAHA, also known as Vorinostat, on GABA uptake in “heterozygous” variant conditions. Both compounds are known to increase gene transcription through chromatin remodeling.

HEK293T cells co‐transfected with WT and variant GAT‐1 constructs were treated with either butyrate [2 mM] or SAHA [2.5 μM] for 24 h. GABA uptake was measured using ^3^H GABA uptake assays and normalized to the vehicle‐treated baseline for each variant (self = 1). Butyrate treatment resulted in a modest increase ranging from 7.4%–17.8**%** relative to untreated across all 32 variants (Figure 3B). Although the increase reached statistical significance in many conditions, the magnitude of change was small, and the response was highly variable. Significant increases in GABA uptake were observed in most variants, including S295L (***p < 0.001), G234S (*p < 0.05), A305T (*p < 0.05), A334P (*p < 0.05), A357V (*p < 0.05), and G566H (**p < 0.01), among others. However, the overall rescue remained well below that observed with PBA or TUDCA. Only one variant, G232V, failed to reach statistical significance.

In contrast, SAHA treatment had minimal and inconsistent impact on GABA uptake across the variant panel. The effect on uptake ranged from −5.2%–13.4% relative to untreated (Figure 3C), and no variant reached statistical significance except the non‐functional control condition (pcDNA, ****p < 0.0001). All remaining variants, including those that responded to PBA and TUDCA, failed to show significant increases in uptake following SAHA treatment. These data indicate that pan‐HDAC inhibition is insufficient to rescue transporter function in the context of *SLC6A1* variants.

### 3.4 ER Stress Modulators, in Addition to PBA and TUDCA, Increased GABA Uptake and GAT‐1 Protein Expression in Disease‐Associated Variants

To further clarify whether reducing ER stress alone is sufficient to improve GAT‐1 function, we also included salubrinal in these experiments. Salubrinal is a selective inhibitor of ER stress‐induced apoptosis that blocks eIF2α dephosphorylation and serves as a mechanistic counterpoint to HDAC inhibitors and pharmacochaperones [21]. To compare the effects of pharmacochaperoning and ER stress modulation on GAT‐1 function, we tested PBA, TUDCA, and salubrinal across multiple *SLC6A1* variants. Unlike pharmacochaperones, which directly stabilize folding intermediates and facilitate ER export, salubrinal does not interact with misfolded proteins but instead modulates the unfolded protein response. This distinction allowed us to evaluate whether alleviating ER stress alone is sufficient to restore transporter function in the absence of direct chaperoning activity.

**FIGURE 4:**
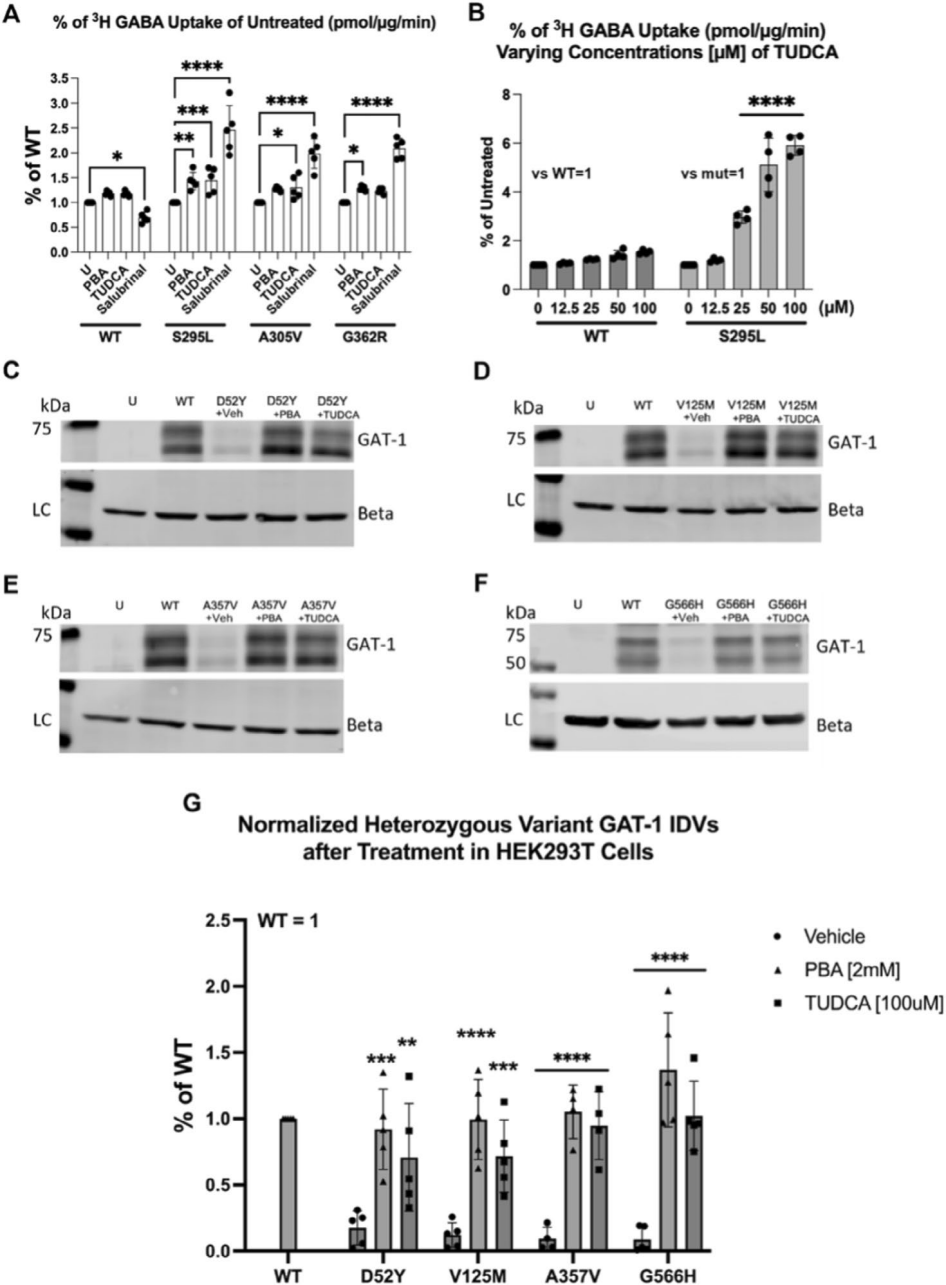
Comparative Effects of Pharmacochaperones and ER Stress Modulators on GABA uptake Function and expression of the mutant transporter 1. (A) ^3^H GABA uptake assay following 24‐h treatment with PBA [2 mM], TUDCA [100 μM], or Salubrinal [15 μM] in WT and heterozygous HEK293T cells expressing S295L, A305V, or G362R variants. Two‐way ANOVA with Dunnett's post hoc test was used to compare drug‐treated to untreated conditions. PBA and TUDCA had no effect in WT cells, while Salubrinal modestly decreased uptake (*p = 0.0342). In S295L cells, PBA (**p = 0.0012), TUDCA (***p = 0.0007), and Salubrinal (****p < 0.0001) all significantly improved uptake, with Salubrinal having the strongest effect. In A305V, TUDCA (*p = 0.0300) and Salubrinal (****p < 0.0001) significantly increased uptake; PBA showed a non‐significant trend (*p* = 0.0587). In G362R, only PBA (*p = 0.0459) and Salubrinal (****p < 0.0001) were effective. (B) Titration of TUDCA (12.5–100 μM) in WT and S295L cells confirmed 100 μM as the optimal concentration, with significant increases in uptake observed at 25–100 μM (****p < 0.0001) in S295L but not in WT. (C–F) Western blot analysis of total GAT‐1 expression in untreated, PBA‐treated [2 mM], and TUDCA‐treated [100 μM] conditions. Β‐Actin (Beta) was used as a loading control. Variants assessed included D52Y (C), V125M (D), A357V (E), and G566H (F). (G) Densitometric quantification showed that both PBA and TUDCA significantly increased total GAT‐1 protein across all tested variants. In D52Y, PBA (***p = 0.0001) had a stronger effect than TUDCA (**p = 0.0053). In V125M, PBA (****p < 0.0001) and TUDCA (**p = 0.0018) both increased expression. A357V and G566H showed robust increases with both treatments (****p < 0.0001). These results support a shared pharmacochaperone mechanism for PBA and TUDCA and suggest that ER stress modulation by Salubrinal may offer additional benefit for select variants.

We began by comparing the effects of each compound on GABA uptake in HEK293T cells expressing either WT or heterozygous GAT‐1 variants (S295L, A305V, or G362R). Cells were treated for 24 h with PBA [2 mM], TUDCA [100 μM], or salubrinal [15 μM], followed by ^3^H GABA uptake quantification (Figure 4A). *In WT cells, salubrinal resulted in a modest but statistically significant decrease in GABA uptake (p = 0.0342), while PBA and TUDCA had no significant effect*. In S295L cells, all three compounds significantly improved uptake: PBA (**p = 0.0012), TUDCA (***p = 0.0007), and salubrinal (****p < 0.0001). In A305V cells, TUDCA (*p = 0.0300) and salubrinal (****p < 0.0001) significantly increased uptake, whereas PBA did not reach significance (*p* = 0.0587). In G362R cells, uptake improved following PBA (*p = 0.0459) and salubrinal (****p < 0.0001), but not TUDCA (*p* = 0.1209). These results suggest that both pharmacochaperoning and ER stress modulation can partially rescue function, although the effect is variant‐dependent.

To validate the working concentration of TUDCA used in these experiments, we conducted a dose–response experiment using “heterozygous” WT and S295L cells treated with TUDCA [0–100 μM] for 24 h. TUDCA had no significant effect on uptake in WT cells at any dose (Figure 4B). In contrast, the patient variant is more sensitive; S295L cells showed a dose‐dependent increase in uptake, with 25 μM, 50 μM, and 100 μM producing significant improvements compared to untreated (****p < 0.0001), confirming that 100 μM is an effective concentration for maximal rescue.

To determine whether functional rescue correlates with increased GAT‐1 protein levels, we determined the GAT‐1 expression in heterozygous conditions across four representative variants: D52Y, V125M, A357V, and G566H. GAT‐1 was detected using a GAT‐1‐specific antibody and normalized to β‐actin loading control (LC) (Figure 4C–F). Densitometry quantification revealed that both PBA and TUDCA significantly increased total GAT‐1 protein in all four variants (Figure 4G). In D52Y cells, PBA (***p = 0.0001) and TUDCA (**p = 0.0053) significantly elevated expression compared to untreated. In V125M, A357V, and G566H cells, both compounds significantly increased protein levels (***p < 0.0001 for all comparisons), supporting their role as pharmacochaperones.

### 3.5 HDAC Inhibitors Failed to Rescue GABA Uptake and Disproportionately Increased Immature GAT‐1 Protein

**FIGURE 5:**
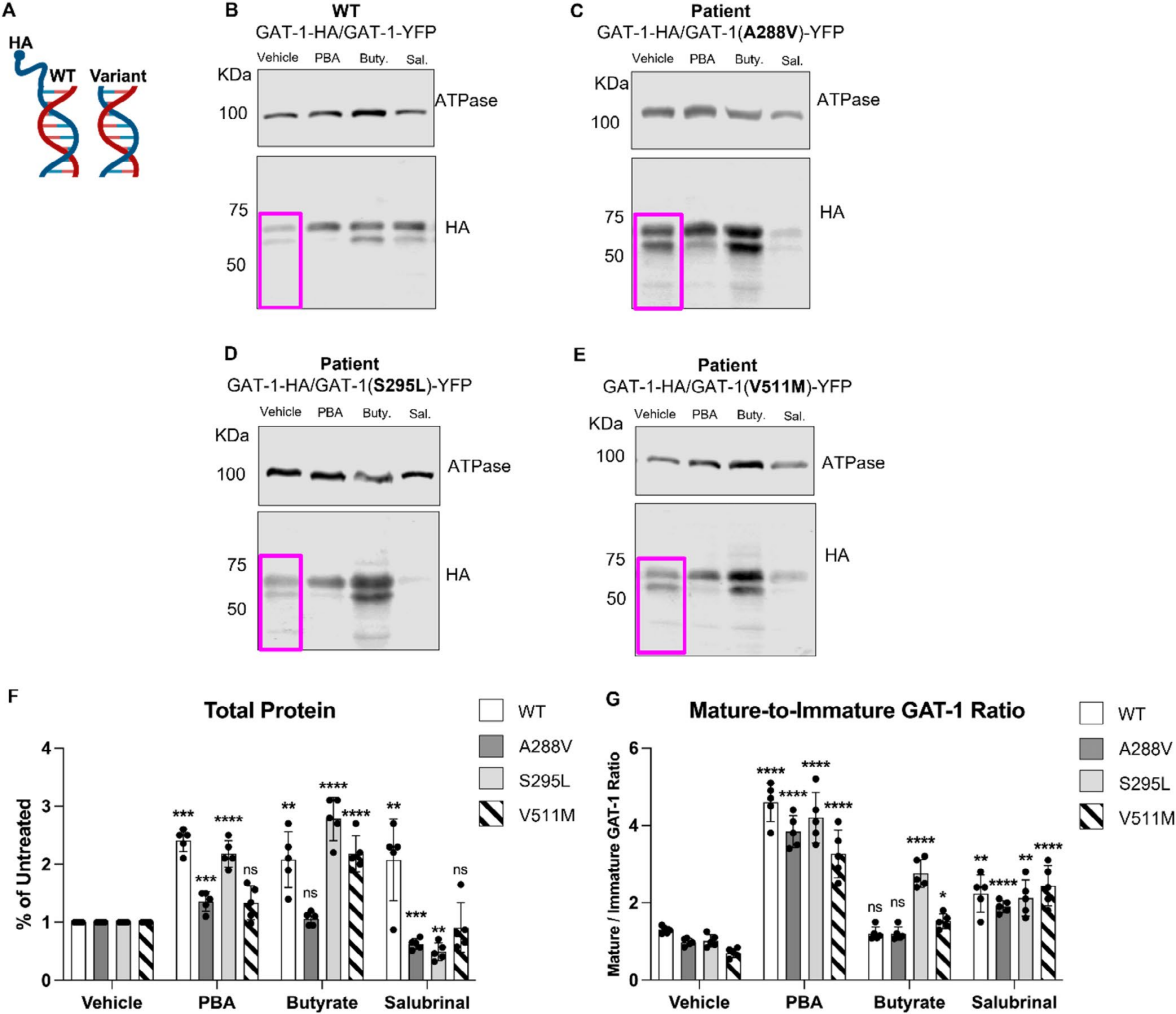
PBA Enhances GAT‐1 Protein Maturation and Reduces the Immature Protein in Heterozygous Variant Conditions. (A) Schematic of heterozygous transfection strategy. Wild‐type (WT) GAT‐1 cDNA was tagged with an HA epitope for selective detection, and mutant GAT‐1 cDNA was tagged with YFP. Equal amounts (1:1) of WT and variant plasmids were co‐transfected to model heterozygous conditions. (B–E) Western blot analysis of GAT‐1‐HA/variant‐GAT‐1‐YFP expression in HEK293T cells following 24‐h treatment with vehicle, PBA [2 mM], butyrate [2 mM], or salubrinal [50 μM]. HA antibody was used to selectively detect WT GAT‐1. ATPase was used as a loading control. WT transfections (B) showed a single mature band, whereas variant transfections with A288V (C), S295L (D), and V511M (E) showed multiple lower molecular weight bands, indicating accumulation of immature, ER‐retained WT protein. (F) Quantification of total HA‐tagged GAT‐1 protein levels. Total protein was normalized to ATPase and compared to vehicle control using one‐way ANOVA with Dunnett's multiple comparisons test. WT: PBA (***p = 0.0003), butyrate (**p = 0.0033), salubrinal (**p = 0.0035); A288V: PBA (***p = 0.0003), butyrate (ns, *p* = 0.6878), salubrinal (***p = 0.0002); S295L: PBA (****p < 0.0001), butyrate (****p < 0.0001), salubrinal (**p = 0.0084); V511M: PBA (ns, *p* = 0.2269), butyrate (****p < 0.0001), salubrinal (ns, *p* = 0.9353). (G) Ratio of mature GAT‐1 (Band 1) to immature forms (Bands 2 and 3). Higher values reflect improved trafficking and maturation of WT GAT‐1. Ratios were analyzed by one‐way ANOVA with Dunnett's multiple comparisons test against the vehicle. WT: PBA (****p < 0.0001), butyrate (ns, *p* = 0.9572), salubrinal (**p = 0.0021); A288V: PBA (****p < 0.0001), butyrate (ns, *p* = 0.3665), salubrinal (****p < 0.0001); S295L: PBA (****p < 0.0001), butyrate (****p < 0.0001), salubrinal (**p = 0.0034); V511M: PBA (****p < 0.0001), butyrate (*p = 0.0190), salubrinal (****p < 0.0001) Data are shown as mean ± SEM from 4 to 5 biological replicates.

To directly assess the trafficking dynamics of WT GAT‐1 protein in the presence of variant alleles, we co‐transfected the HA‐tagged wildtype and the YFP‐tagged variant GAT‐1 at a 1:1 ratio (Figure 5A). In WT‐only conditions (Figure 5B), a single upper band was observed, consistent with fully mature, glycosylated GAT‐1. In contrast, cells co‐expressing A288V, S295L, or V511M variants (Figure 5C–E) exhibited prominent lower molecular weight bands corresponding to immature, ER‐retained forms of the WT protein, indicating impaired trafficking and delayed maturation. This confirms that misfolded GAT‐1 variants hinder the processing of WT protein, likely by saturating ER quality control systems.

Treatment with PBA [2 mM] for 24 h significantly increased total WT GAT‐1 protein levels in WT‐only (***p = 0.0003), A288V (***p = 0.0003), and S295L (****p < 0.0001) conditions (Figure 5F). In V511M cells, PBA showed a modest, non‐significant increase (*p* = 0.2269). Butyrate [2 mM] also increased total GAT‐1 in WT (**p = 0.0033) and S295L (****p < 0.0001) conditions but had no effect in A288V (*p* = 0.6878) and V511M (****p < 0.0001). Salubrinal [50 μM] significantly increased total protein levels in WT (**p = 0.0035), A288V (***p = 0.0002), and S295L (**p = 0.0084), but had no effect in V511M (*p* = 0.9353). These results confirm that PBA consistently enhances WT protein expression, but butyrate and salubrinal display inconsistent or variant‐specific effects.

We then calculated the ratio of mature GAT‐1 (Band 1) to immature forms (Bands 2 and 3), based on our previous characterization of their respective molecular weights (Figure 5G) [9]. Higher ratios reflect successful folding, ER export, and glycosylation. In all conditions, PBA significantly increased the mature‐to‐immature ratio, indicating improved trafficking: WT (****p < 0.0001), A288V (****p < 0.0001), S295L (****p < 0.0001), and V511M (****p < 0.0001). Butyrate failed to increase this ratio in WT (*p* = 0.9572), A288V (*p* = 0.3665), and showed only a weak effect in V511M (*p = 0.0190). In contrast, butyrate significantly increased the total amount of immature protein, suggesting that it drives non‐specific protein accumulation rather than productive trafficking. Salubrinal significantly increased trafficking ratios in WT (**p = 0.0021), A288V (****p < 0.0001), S295L (**p = 0.0034), and V511M (****p < 0.0001), although to a lesser extent than PBA.

### 3.6 PBA Restored GABA Uptake in Slc6a1^+/A288V^ and Slc6a1^+/S295L^ Knock‐In Mice, While Butyrate Failed to Rescue Transporter Function in the Synaptosomes

To evaluate the in vivo efficacy of pharmacological agents on GABA transporter function, we conducted ^3^H GABA uptake assays using crude synaptosomes isolated from the forebrain of Slc6a1^+/A288V^ and Slc6a1^+/S295L^ knock‐in mice. Mice were treated with daily intraperitoneal (i.p.) injections of either PBA [100 mg/kg] or butyrate [100 mg/kg] for 7 consecutive days. Uptake was measured in raw counts per minute (CPM), and statistical comparisons were made using unpaired *t*‐tests.

At baseline, both Slc6a1^+/A288V^ and Slc6a1^+/S295L^ knock‐in mice exhibited significantly reduced synaptosomal GABA uptake compared to their respective WT controls, consistent with impaired transporter function.

**FIGURE 6:**
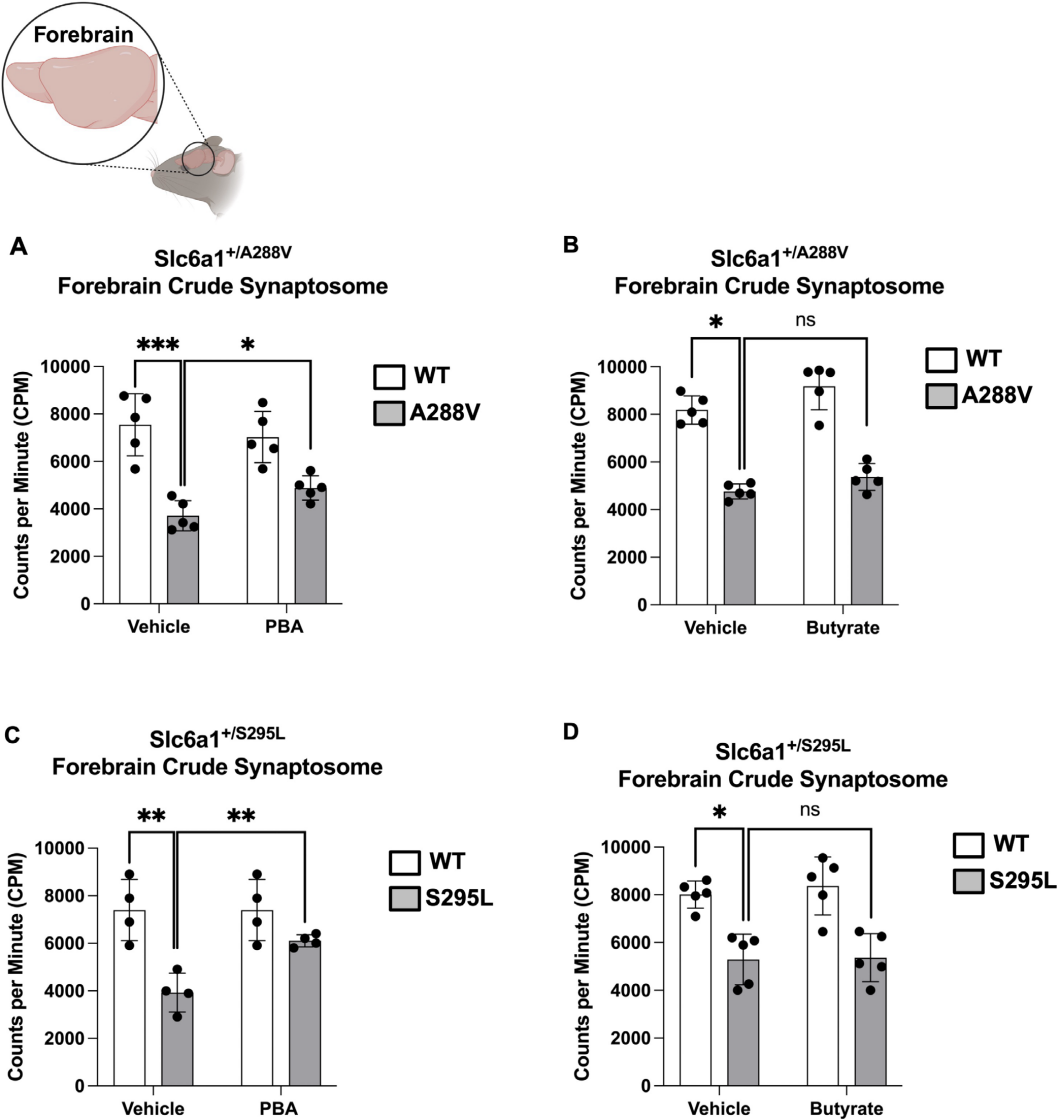
Rescue of GABA Uptake by PBA in Forebrain Synaptosomes from *SLC6A1* Variant Mice. (A) ^3^H GABA uptake in crude synaptosomes from Slc6a1^+/A288V^ knock‐in mice. GABA uptake was quantified in raw counts per minute (CPM) in forebrain synaptosomes. Significant reduction in uptake was observed in vehicle‐treated Slc6a1^+/A288V^ mice compared to WT, while butyrate treatment (100 mg/kg, i.p., daily for 7 days) did not significantly increase uptake compared to baseline Slc6a1^+/A288V^. Statistical analysis was performed using unpaired *t*‐tests: WT vs. Slc6a1^+/A288V^ baseline (****p < 0.0001); A288V baseline vs. butyrate‐treated (*p* = 0.0680). (B) ^3^H GABA uptake in crude synaptosomes from Slc6a1^+/A288V^ knock‐in mice. GABA uptake was quantified in raw counts per minute (CPM) in forebrain synaptosomes. Significant reduction was observed in untreated Slc6a1^+/A288V^ mice compared to WT, while PBA treatment (100 mg/kg, i.p., daily for 7 days) significantly restored uptake. Statistical analysis was performed using unpaired *t*‐tests: WT vs. Slc6a1^+/A288V^ baseline (***p = 0.0004); A288V baseline vs. PBA‐treated (*p = 0.0124). (C) ^3^H GABA uptake in crude synaptosomes from Slc6a1^+/S295L^ knock‐in mice. GABA uptake was quantified in raw counts per minute (CPM) in forebrain synaptosomes. Significant reduction in uptake was observed in untreated S295L mice compared to WT, while butyrate treatment (100 mg/kg, i.p., daily for 7 days) did not significantly improve uptake compared to baseline S295L. Statistical analysis was performed using unpaired *t*‐tests: WT vs. *SLC6A1* ^+/S295L^ baseline (***p = 0.0010); S295L baseline vs. butyrate‐treated (*p* = 0.9121). (D) ^3^H GABA uptake in crude synaptosomes from Slc6a1^+/S295L^ knock‐in mice. GABA uptake was quantified in raw counts per minute (CPM) in forebrain synaptosomes. Significant reduction in uptake was observed in untreated S295L mice compared to WT, while PBA treatment (100 mg/kg, i.p., daily for 7 days) significantly increased uptake compared to baseline Slc6a1^+/S295L^. Statistical analysis was performed using unpaired *t*‐tests: WT vs. Slc6a1^+/S295L^ baseline (**p = 0.0039); Slc6a1^+/S295L^ baseline vs. PBA‐treated (**p = 0.0023).

In Slc6a1^+/A288V^ mice, GABA uptake was reduced by 50.8% compared to WT (***p < 0.0001) (Figure 6A). Butyrate treatment did not result in a significant increase in uptake (*p* = 0.0680). In contrast, PBA significantly increased uptake by 31.5% compared to baseline (*p* = 0.0124), restoring 64.7% of WT transporter activity (Figure 6B). Similarly, Slc6a1^+/S295L^ mice showed a 46.9% reduction in uptake compared to WT (***p = 0.0010) (Figure 6C). Butyrate treatment did not result in a significant increase in uptake (*p* = 0.9121). In contrast, PBA elicited a 55.4% increase in uptake over baseline (*p* = 0.0023), corresponding to 82.4% of WT function (Figure 6D).

Untreated variant mice in both genotypes remained significantly different from WT following the 7‐day treatment course (A288V: ***p = 0.0004; S295L: **p = 0.0039), highlighting the pathogenic impact of these variants and validating the use of synaptosomal uptake as a measure of functional restoration ex vivo.

### 3.7 Chaperone Overexpression and BiP Activation Enhanced GAT‐1 Function and Protein Expression in the Cells Expressing the WT GAT‐1 and GAT‐1(S295L)

Previous data demonstrated that PBA improves GAT‐1 trafficking and expression by promoting proper protein folding, suggesting its pharmacochaperoning effect. To directly test the role of endogenous chaperone systems in regulating GAT‐1 function, we evaluated whether overexpression or pharmacological activation of ER‐resident chaperones could enhance transporter maturation and function. HEK293T cells were co‐transfected with either GAT‐1 WT or GAT‐1(S295L) and one of three constructs: Empty vector (pcDNA), BiP, or Calnexin. BiP (also known as GRP78) and Calnexin are both ER‐localized chaperones involved in protein quality control and folding within the protein secretory pathway in the ER.

**FIGURE 7:**
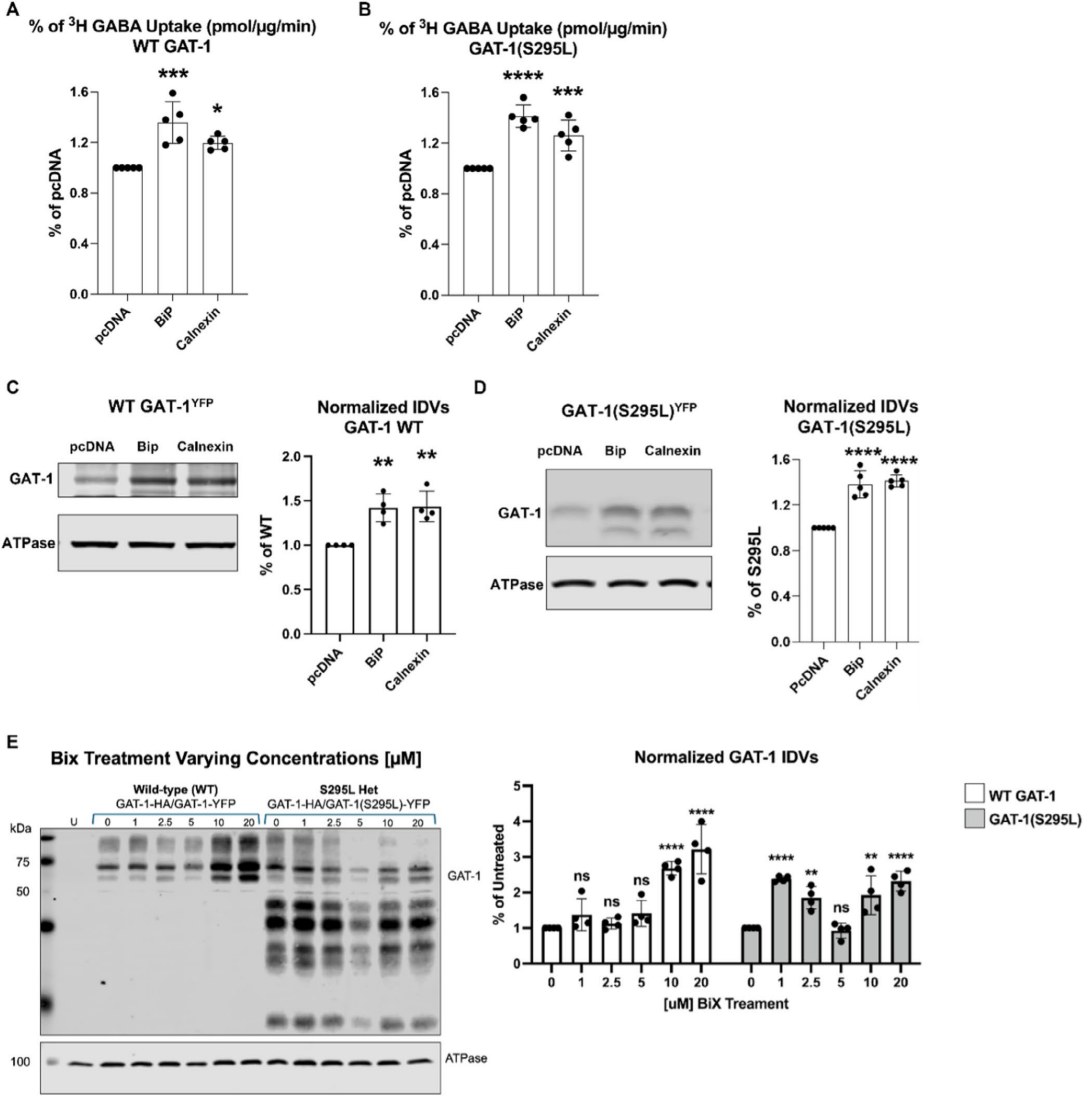
Chaperone Overexpression and Bix Treatment Increased GABA Uptake and GAT‐1 Protein Expression. (A) ^3^H GABA uptake in HEK293T cells co‐transfected with GAT‐1 WT and either empty vector (pcDNA), BiP, or Calnexin at a 1:1 ratio. GABA uptake was significantly increased in cells co‐expressing BiP or Calnexin compared to pcDNA control. Statistical analysis was performed using one‐way ANOVA with Dunnett's multiple comparisons test: PcDNA vs. BiP (***p = 0.0002); pcDNA vs. Calnexin (*p = 0.0169). (B) ^3^H GABA uptake in HEK293T cells co‐transfected with GAT‐1(S295L) and either pcDNA, BiP, or Calnexin at a 1:1 ratio. GABA uptake was significantly increased in cells co‐expressing BiP or Calnexin compared to S295L/pcDNA. Statistical analysis was performed using one‐way ANOVA with Dunnett's multiple comparisons test: PcDNA vs. BiP (****p < 0.0001); pcDNA vs. Calnexin (***p = 0.0010). (C) Western blot analysis of GAT‐1 WT protein levels in HEK293T cells co‐expressed with pcDNA, BiP, or Calnexin. ATPase was used as a loading control. Total GAT‐1 protein was significantly increased by both chaperones compared to the pcDNA control. Statistical analysis was performed using one‐way ANOVA with Dunnett's multiple comparisons test: PcDNA vs. BiP (**p = 0.0031); pcDNA vs. Calnexin (**p = 0.0024). (D) Western blot analysis of GAT‐1(S295L) protein levels in HEK293T cells co‐expressed with pcDNA, BiP, or Calnexin. ATPase was used as a loading control. Both BiP and Calnexin significantly increased total GAT‐1(S295L) expression compared to pcDNA control. Statistical analysis was performed using one‐way ANOVA with Dunnett's multiple comparisons test: PcDNA vs. BiP (****p < 0.0001); pcDNA vs. Calnexin (****p < 0.0001). (E) Western blot analysis of GAT‐1 protein expression in HEK293T cells treated with increasing concentrations of Bix [0 (vehicle only), 1, 2.5, 5, 10, 20 μM] for 24 h. Protein levels were assessed in both WT and S295L backgrounds. ATPase was used as a loading control. Statistical analysis was performed using one‐way ANOVA with Dunnett's multiple comparisons test relative to the vehicle (0 μM) condition. For WT: 0 vs. 1 μM (*p* = 0.5196); 0 vs. 2.5 μM (*p* = 0.9867); 0 vs. 5 μM (*p* = 0.4328); 0 vs. 10 μM (****p < 0.0001); 0 vs. 20 μM (****p < 0.0001). For S295L: 0 vs. 1 μM (****p < 0.0001); 0 vs. 2.5 μM (**p = 0.0033); 0 vs. 5 μM (*p* = 0.9963); 0 vs. 10 μM (**p = 0.0014); 0 vs. 20 μM (****p < 0.0001).

Overexpression of either BiP or Calnexin significantly increased GABA uptake in both WT and S295L conditions. In cells expressing GAT‐1 WT, BiP (***p = 0.0002) and Calnexin (*p = 0.0169) significantly enhanced GABA uptake compared to pcDNA controls (Figure 7A). In the S295L variant, which is known to display severe trafficking deficits and dominant negative effects, both BiP (****p < 0.0001) and Calnexin (***p = 0.0010) also significantly improved uptake relative to pcDNA (Figure 7B). BiP overexpression increased GABA uptake in the cells expressing both the WT (36%) and S295L variants (41%). These findings indicate that increasing endogenous chaperone availability is sufficient to enhance transporter function, even in the presence of pathogenic variants. Similar findings were obtained for the GAT‐1 expression when the WT or S295L variant cDNAs were cotransfected with both BiP (**p = 0.0031) and Calnexin (**p = 0.0024) relative to pcDNA (Figure 7C,D). BiP overexpression increased GAT‐1 protein expression by 42% in the WT condition and 39% in the S295L condition. Notably, S295L lanes exhibited multiple lower molecular weight bands consistent with ER‐retained or immature forms of the transporter, consistent with prior findings of impaired trafficking in this variant ^9.15^.

To pharmacologically enhance endogenous chaperone activity, we treated WT and S295L cells with increasing concentrations of Bix, a small‐molecule BiP activator, for 24 h. WB analysis revealed that BiX increased GAT‐1 protein expression in a dose‐dependent manner. In WT cells, significant increases were observed at 10 μM and 20 μM (****p < 0.0001 for both) (Figure 7E). In S295L cells, Bix also increased GAT‐1 expression at 1 μM (****p < 0.0001), 2.5 μM (**p = 0.0033), 10 μM (**p = 0.0014), and 20 μM (****p < 0.0001), confirming the effectiveness of BiP activation even in the context of misfolded or trafficking‐deficient variant protein. No significant increase was observed at 5 μM in either condition.

### 3.8 Drug Treatment and Seizure Endpoints in Slc6a1^+/A288V^ and Slc6a1^+/S295L^ Knock‐In Mice

**FIGURE 8:**
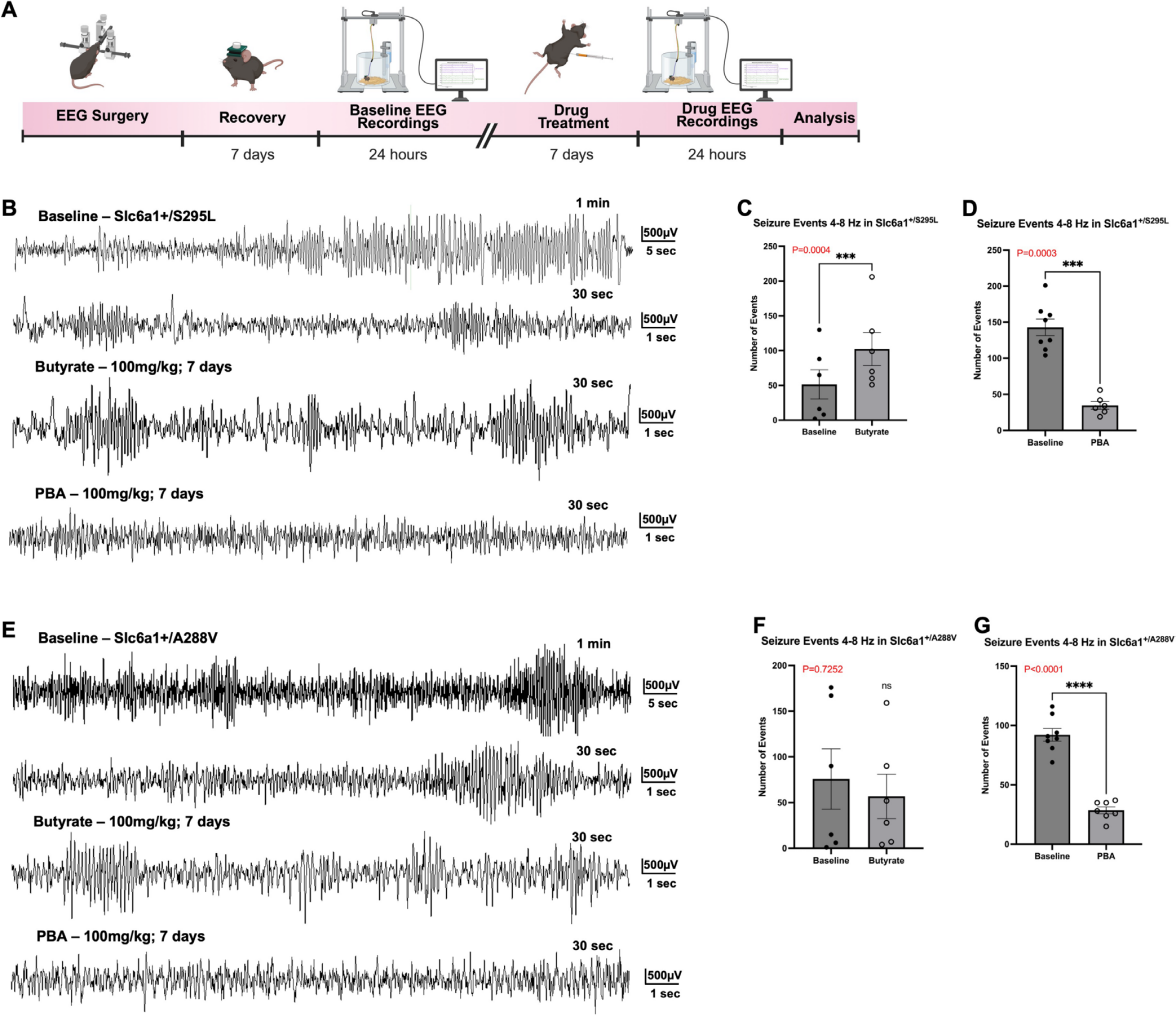
HDAC Treatment and Seizure Endpoints in Slc6a1^*+/A288V*^ and Slc6a1^*+/S295L*^ Knock‐in Mice. (A) Schematic diagram depicting EEG head mount implantation and recording timeline. Mice underwent EEG head mount surgery, followed by a 7‐day recovery period, before baseline EEG recordings were obtained over 24 h. After baseline recordings, mice were treated with butyrate (100 mg/kg, i.p., daily for 7 days), followed by a second 24‐h EEG recording session to assess the effects of HDAC inhibition on seizure frequency and duration. (B) Representative EEG traces from A288V heterozygous mice, displaying baseline seizure events before butyrate treatment and EEG traces following butyrate administration. These recordings allow for qualitative assessment of seizure frequency and duration changes following HDAC inhibition. (C) Quantification of seizure events in Slc6a1^+/S295L^ mice (4–8 Hz frequency range). EEG recordings were analyzed to compare seizure frequency between baseline and butyrate‐treated conditions. A significant increase in seizure frequency was observed following butyrate treatment (100 mg/kg, i.p., daily for 7 days), as determined by a paired *t*‐test (*p = 0.0119). (D) Representative EEG traces from S295L heterozygous mice, showing baseline seizure events before butyrate treatment and EEG traces following butyrate administration. The EEG traces illustrate absence‐like spike–wave discharges (SWDs) and myoclonic jerks observed in untreated baseline recordings compared to post‐treatment seizure activity. (E) Quantification of seizure events in Slc6a1^+/S295L^ mice (4–8 Hz frequency range). A significant reduction in seizure frequency was observed following PBA treatment (100 mg/kg, i.p., daily for 7 days), as determined by a paired *t*‐test (***p = 0.0003). (F) Quantification of seizure events in Slc6a1^+/A288V^ mice (4–8 Hz frequency range). EEG analysis revealed a non‐significant trend toward reduced seizure frequency following butyrate treatment (100 mg/kg, i.p., daily for 7 days), as determined by a paired *t*‐test (*p* = 0.1046). (G) Quantification of seizure events in Slc6a1^+/A288V^ mice (4–8 Hz frequency range). PBA treatment (100 mg/kg, i.p., daily for 7 days) resulted in a highly significant reduction in seizure frequency compared to baseline, as determined by a paired *t*‐test (****p < 0.0001).

To assess the impact of pharmacological intervention on seizure burden, we conducted 24‐h EEG recordings in heterozygous Slc6a1^+/S295L^ and Slc6a1^+/A288V^ knock‐in mice. Each mouse underwent surgical implantation of an EEG head mount, followed by a 7‐day recovery period prior to baseline recordings. Mice were then treated daily with either butyrate or PBA [100 mg/kg, i.p., for 7 days], followed by a second 24‐h recording to compare seizure frequency across treatment conditions (Figure 8A). Seizure events were quantified using Sirenia Seizure Pro and Clampfit analysis, with a focus on rhythmic discharges within the 4–8 Hz range as in our previous studies [15, 24].

In *Slc6a1* ^*+/S295L*^ mice, butyrate treatment significantly increased the number of seizure events compared to baseline (*p* = 0.0119) (Figure 8C). Representative EEG traces revealed prominent spike–wave discharges and myoclonic bursts that were more frequent following HDAC inhibition (Figure 8B), suggesting that butyrate not only failed to mitigate seizure activity but potentially worsened disease phenotype in this genotype. In contrast, PBA treatment led to a significant reduction in seizure frequency in the same Slc6a1^+/S295L^ mice (*p = 0.0003) (Figure 8D), highlighting a divergent therapeutic response between the two compounds. These findings reinforce that pharmacochaperoning via PBA alleviates seizure burden, while HDAC inhibition through butyrate may exacerbate network instability in certain variants.

In *Slc6a1* ^*+/A288V*^ mice, butyrate treatment did not significantly alter seizure frequency, although a mild trend toward reduction was observed (*p* = 0.1046) (Figure 8F). EEG traces showed no qualitative evidence of improvement (Figure 8E), and variability between individual mice may underlie the lack of statistical significance. Notably, PBA administration produced a robust and highly significant reduction in seizure events in Slc6a1^+/A288V^ mice (**p < 0.0001) (Figure 8G), mirroring the therapeutic efficacy observed in the Slc6a1^+/S295L^ model.

## 4 Discussion

### 4.1 *SLC6A1* Variants Cause Loss of Function via ER Retention and Protein Trafficking Deficits

SLC6A1 variants lead to profound GABA transporter dysfunction, disrupting inhibitory neurotransmission through a combination of impaired trafficking, altered protein folding, and reduced transporter activity. These deficits result in marked reductions in GABA uptake, which contribute directly to the neurological symptoms observed in affected individuals. Across all 32 variants tested in this study, we observed significant reductions in GABA uptake in both homozygous and heterozygous models, supporting loss of function as the central pathogenic mechanism.

While ER retention and trafficking deficits are well established for many SLC6A1 variants, prior studies have demonstrated that certain variants, including R44Q, R44W, G232V, and G297R, can reach the cell surface, indicating that not all variants uniformly exhibit ER retention [27, 28]. Thus, the relative contribution of trafficking defects vs. intrinsic functional impairment is variant dependent. In addition, for variants not previously characterized, the observed functional deficits may reflect impaired trafficking, intrinsic transporter dysfunction, or a combination of both.

Importantly, variant GAT‐1 may become retained or improperly processed in the ER and interfere with the trafficking of WT GAT‐1 in heterozygous conditions, contributing to reduced overall transporter function.

### 4.2 Upregulation of Chaperones Is Sufficient to Restore GAT‐1 Function and Expression

To further investigate the role of ER proteostasis in *SLC6A1* pathogenesis, we tested whether boosting chaperone activity was sufficient to restore transporter function and expression. We found that co‐expression of either BiP or Calnexin with GAT‐1, both in WT and S295L conditions, significantly increased GABA uptake and total protein levels. In GAT‐1(S295L)‐expressing cells, where trafficking is severely impaired, chaperone co‐expression rescued both function and expression, highlighting the importance of the ER folding environment in determining transporter fate.

These findings were further corroborated by pharmacological activation of BiP using BiX, a small‐molecule modulator of the ER chaperone response. BiX significantly increased GAT‐1 protein levels in a concentration‐dependent manner in the cells expressing both WT GAT‐1 and GAT‐1(S295L). However, persistent accumulation of immature, lower molecular weight bands in the GAT‐1(S295L) lanes suggested that Bix improves protein stability but does not fully resolve trafficking impairments in severe variants. These findings indicate that endogenous chaperone insufficiency may play a role in *SLC6A1*‐mediated pathophysiology, supporting the concept that enhancing chaperone capacity is sufficient to partially stabilize and increase variant transporter expression.

This aligns with broader principles of protein misfolding disorders, where variant proteins become trapped in the ER and disrupt cellular homeostasis. Similar mechanisms have been observed in GABA_A_ receptor variants, cystic fibrosis, and other neurogenetic disorders, reinforcing the idea that correcting ER trafficking deficits is a critical therapeutic goal [10, 11, 12, 13, 24, 29]. By demonstrating that GAT‐1 variants follow a common misfolding and ER retention pathway, this study provides new mechanistic insight into *SLC6A1* disorders and identifies pharmacological chaperoning as a promising intervention.

### 4.3 Pharmacochaperones Outperform HDAC Inhibitors in Restoring GAT‐1 Function

PBA treatment significantly increased GABA uptake across all 32 tested *SLC6A1* variants, confirming its role as a pharmacochaperone that facilitates proper folding and trafficking of GAT‐1 to the plasma membrane. TUDCA, another pharmacochaperone, exhibited similar functional rescue, further supporting that targeting protein stability and trafficking is a viable therapeutic strategy. The comparable rescue profiles of PBA and TUDCA, combined with the absence of benefit from HDAC inhibitors butyrate and SAHA, provide direct experimental evidence that PBA's therapeutic effect is primarily driven by pharmacochaperoning rather than histone deacetylase inhibition. This interpretation is strengthened by the fact that TUDCA, which lacks HDAC inhibitory activity, achieved a similar magnitude and pattern of variant rescue as PBA. The similarity in efficacy profiles and the consistent direction of response across variants suggest that PBA may exert its effects through a post‐translational mechanism, potentially by enhancing folding and trafficking of misfolded GAT‐1 to the cell surface.

In contrast, HDAC inhibition via SAHA and butyrate failed to restore GABA uptake to WT levels or alleviate ER stress, reinforcing that PBA's therapeutic mechanism is distinct from transcriptional regulation or increasing the total transporter protein. HDAC inhibitors broadly alter gene expression, leading to increased global protein synthesis [17, 22]. This is consistent with our observation of the increased GAT‐1 in butyrate‐treated cells. However, this indiscriminate effect is not inherently beneficial for *SLC6A1* variant‐mediated disorders, as it results in the synthesis of both functional and misfolded nonfunctional proteins. This collateral increase in misfolded proteins exacerbates ER stress, further overwhelming the proteostasis network and worsening trafficking deficits rather than alleviating them.

While some HDAC inhibitors have demonstrated short‐term or context‐specific benefits in other neurological disorders, including improved seizure thresholds in tuberous sclerosis, delayed epileptogenesis in temporal lobe epilepsy, and restored memory performance in Alzheimer's disease models, these effects are often transient and symptom‐limited [30, 31, 32, 33]. Modest improvements in motor coordination have also been reported in Huntington's disease models following HDAC1/3 inhibition, though without broader behavioral or neuroprotective benefit [34]. The complex and developmentally persistent nature of *SLC6A1* variant‐mediated epilepsy necessitates durable and mechanism‐targeted treatment strategies.

Data from this study demonstrate that PBA improves trafficking of WT GAT‐1 protein in the context of heterozygous *SLC6A1* variants by increasing the proportion of mature, correctly processed functional transporters. In contrast, butyrate promotes total protein expression by increasing both the mature and immature proteins, potentially leading to ER accumulation. These findings from PBA and butyrate support that targeting protein folding and trafficking, rather than broad epigenetic regulation, is the superior therapeutic approach for restoring GAT‐1 function. Notably, PBA treatment significantly increased GABA uptake not only in vitro but also ex vivo, rescuing synaptosomal uptake in *Slc6a1* ^*+/A288V*^ and *Slc6a1* ^*+/S295L*^ knock‐in mice. Pharmacochaperoning directly enhances protein misfolding and reduces ER retention, while HDAC inhibition increases total protein and likely increases ER stress. Thus, PBA and related chaperone‐based strategies represent promising therapeutic candidates for *SLC6A1* variant‐mediated disorders by enabling functional GABA transporter recovery and restoring inhibitory balance in the CNS.

Because experiments were conducted under heterozygous conditions (WT + variant), pharmacological effects observed in this study may partially reflect modulation of WT GAT‐1 in addition to rescue of variant protein. This represents an inherent limitation of disease‐relevant heterozygous models but also reflects the genetic context of *SLC6A1*‐related disorders. Importantly, the variant‐dependent differences in response to pharmacochaperones and HDAC inhibitors observed across conditions support a mutation‐specific component to functional rescue beyond WT modulation.

### 4.4 The Therapeutic Effect of HDAC Inhibition in Other Epilepsies

Recent studies have explored the potential of HDAC inhibition as a therapeutic strategy for epilepsy‐related pathology, including neuroprotection and cognitive preservation. Notably, Taylor et al. [35] demonstrated that HDAC inhibitors such as valproic acid and SAHA improved outcomes in acute seizure models by reducing hippocampal neurodegeneration and preserving cognitive function. Their findings suggest that HDAC inhibition may reduce seizure‐induced neuronal injury through modulation of apoptotic, inflammatory, and oxidative stress pathways in certain cases. These results highlight the therapeutic potential of HDAC inhibitors in neurological disorders marked by excitotoxic injury, neurodegeneration, or progressive neuronal loss [31, 32, 33].

However, the underlying mechanism of disease in *SLC6A1* variant‐mediated disorders differs significantly. Rather than resulting from seizure‐induced neurodegeneration, *SLC6A1* variants cause reduced GAT‐1 due to protein misfolding, ER retention, and trafficking failure. We show that HDAC inhibition with butyrate exhibited modest improvements in GABA uptake in some variants but increased seizure frequency in *Slc6a1* ^*+/S295L*^ mice.

While Taylor et al. [35] provide important evidence that HDAC inhibition may be beneficial in acquired seizure models involving hippocampal neurodegeneration, neuroinflammation, and cognitive decline, their findings reflect a distinct pathophysiological context. Their work contributes meaningfully to understanding how transcriptional modulation can reduce seizure‐induced neurotoxicity in acquired epilepsy settings. In contrast, *SLC6A1* variant‐mediated disorders are characterized by genetic disruption of transporter folding and trafficking, independent of inflammatory processes. PBA consistently achieved greater functional rescue by reducing ER retention, stabilizing variant GAT‐1, and promoting trafficking to the cell surface. These differences in disease mechanism and treatment response help clarify why pharmacochaperoning is more effective in *SLC6A1* variant‐mediated disorders, while HDAC inhibition may hold promise in other forms of epilepsy. Together, these approaches represent distinct but complementary avenues for therapeutic intervention across the broader epilepsy landscape.

### 4.5 Butyrate Fails to Improve Seizure Outcomes in *Slc6a1* ^*+/S295L*^ Mice

In *Slc6a1* ^*+/S295L*^ mice, butyrate treatment led to a significant increase in the number of seizure events (*p* = 0.0119), suggesting a worsening of seizure susceptibility rather than a therapeutic benefit. This increase in seizure burden aligns with the in vitro findings that butyrate does not enhance GABA uptake and may contribute to increased ER stress, which could further impair transporter function. In *Slc6a1* ^*+/A288V*^ mice, butyrate treatment showed a non‐significant decrease in seizure frequency (*p* = 0.1046), suggesting that its effects may vary based on genetic background or variant‐specific factors. However, neither genotype showed any changes in seizure duration, indicating that butyrate does not meaningfully impact overall seizure severity.

These findings provide strong evidence that HDAC inhibition alone is not a viable therapeutic strategy for *SLC6A1* variant disorders. Unlike PBA, which directly enhances protein stability and trafficking, butyrate's mechanism is transcriptionally mediated and lacks the ability to restore functional GAT‐1 at the plasma membrane. Furthermore, the increased seizure frequency in Slc6a1^+/S295L^ mice raises concerns that HDAC inhibition may introduce unintended neuronal hyperexcitability or disrupt compensatory homeostatic mechanisms, especially when administered chronically.

Together, these data demonstrate that butyrate fails to restore synaptic GABA uptake in either *Slc6a1* ^*+/A288V*^ or *Slc6a1* ^*+/S295L*^ mice, despite its known HDAC inhibitory properties. In contrast, PBA significantly enhances transporter function and achieves partial restoration toward WT levels, supporting its role as an effective pharmacochaperone in vivo. These findings confirm that HDAC inhibition alone is insufficient to rescue transporter activity and highlight the translational relevance of PBA as a disease‐modifying therapy for *SLC6A1* variant‐mediated disorders.

In summary, the findings presented in this study reinforce pharmacochaperoning as the primary mechanism by which PBA restores GAT‐1 function, providing strong preclinical evidence that this therapeutic approach directly addresses the underlying cellular deficits in *SLC6A1* variant‐mediated disorders. The findings presented here confirm and extend previous reports, demonstrating that PBA functions as a pharmacochaperone to increase the functional GAT‐1 rather than through epigenetic modulation to increase the total GAT‐1. By demonstrating that PBA enhances protein stability, alleviates ER stress, and promotes proper trafficking, this study resolves a critical question in the field regarding whether PBA functions as a pharmacochaperone or an HDAC inhibitor. This study suggests that increasing functional GAT‐1 and reducing ER‐retained GAT‐1 underlie PBA rescue for *SLC6A1* variants.

## Author Contributions

M.B.D. led the study design and execution, performed experiments, acquired and analyzed data, created figures, and wrote the original draft of the manuscript. K.R. conducted all animal experiments, including drug treatments, EEG recordings, and synaptosome preparations; contributed to data analysis, figure generation, and manuscript review and editing. E.A. and Z.D.S. conducted experiments, acquired data, and contributed to data analysis. W.S. performed experiments, assisted with data validation and analysis, and contributed to manuscript review. All authors reviewed and approved the final version of the manuscript. J.‐Q.K. conceptualized the project, provided methodological and strategic guidance, supervised the research, acquired funding, and contributed to manuscript review and editing.

## Funding

The authors have nothing to report.

## Ethics Statement

All animals and related experiments in this study were approved by the Vanderbilt University IACUC.

## Conflicts of Interest

The authors declare no conflicts of interest.

## Data Availability

Data can be made available upon request.
